# Leveraging context-specific behavioral economic principles to enable patients to change their physical activity patterns

**DOI:** 10.1177/13591053251317320

**Published:** 2025-02-19

**Authors:** Brittany V. Barber, Michael Vallis, George Kephart, Ruth Martin-Misener, Daniel Rainham

**Affiliations:** Dalhousie University, Canada

**Keywords:** behavior change, behavioral economics, behavioral interventions, chronic disease prevention, decision-making processes

## Abstract

This study explores how context-specific behavioral economic principles could be employed to tailor interventions to support patients’ efforts to modify day-to-day routines. Using adapted geo-ethnography techniques, interviews collected in-depth descriptions about facilitators and barriers to physical activity (PA), and contexts influencing decisions about day-to-day activities. Data were analyzed using the Capability, Opportunity, and Motivation - Behaviour (COM-B) model for behavior change and MINDSPACE behavioral economic principles as coding frameworks. Twenty-nine patients (19 men, 10 women) aged 50–79 participated. Findings indicate patients were motivated and capable of increasing PA but were challenged to identify opportunities to adapt day-to-day routines for increasing PA. Patients described disrupting default routines, increasing commitments, changing the messenger, and introducing incentives as potentially useful behavioral economic principles to improve day-to-day decisions about increasing PA. Patients had insight into potential behavioral economic principles, although they were not previously educated, and were valuable partners in developing research and clinic-based behavioral economic intervention strategies.

## Introduction

Cardiovascular diseases (CVD) are the leading cause of death worldwide, accounting for more than 17 million deaths annually (World Health Organization, 2021a), and one-fifth of all deaths in Canada (Statistics Canada, 2020). These deaths are largely preventable through modification of lifestyle risk factors such as smoking, physical inactivity, obesity, poor nutrition, high blood pressure, and excessive consumption of alcohol (Roth et al., 2020; World Health Organization, 2021b). Behavioral interventions for modifying risk factors are critical for reducing risk of CVD or a repeat event.

The effectiveness of interventions to reduce risk of CVD is hindered by poor maintenance of behaviors after an intervention (Graham et al., 2020; McQuoid et al., 2017) when patients no longer have regular contact with healthcare professionals and peers (Bray et al., 2006). Digital tools have been shown to improve intervention effectiveness by sustaining interaction between patients and clinicians, through individualized monitoring and feedback, and by tailoring interventions to acknowledge the unique requirements of patients to maintain behavior change in real-world settings (Inglis et al., 2010; Santo and Redfern, 2020; Schorr et al., 2021). However, the success of these digital tools can be limited when they do not adequately consider the spatiotemporal contexts within which patient health behaviors occur. This additional knowledge is critical to ensure intervention strategies are tailored to incorporate the timing and location that seemingly automatic decisions patients make as part of their daily routines.

Spatiotemporal contexts play an important role in promoting or inhibiting health behaviors (Macintyre et al., 2002). Identifying the unique social and environmental contexts that influence the timing and location of individual health-related activities is critical for tailoring intervention strategies (Barber et al., 2024a). This is especially important considering the powerful influence of associative learning, where environmental cues become conditioned stimuli that elicit behavior (Havermans, 2013). Spatiotemporal contexts of day-to-day health behaviors previously explored by our research team identify when and where routine health activities can be disrupted to increase physical activity (PA) within a clinic-based intervention for CVD (Barber et al., 2024b). We adapted geo-ethnography mixed-method techniques (Matthews et al., 2005; Milton et al., 2015) to develop a data collection and communication tool for collecting geospatial and temporal contexts of patient PA behaviors. This study identified the context and timing of health promoting activities (i.e. walking in neighborhood in morning or early evening) as well as opportunities for disrupting health inhibiting sedentary activities (i.e. watching television in evening; Barber et al., 2024b). Time-use patterns provide a visualization of patients’ unique spatiotemporal sequencing of day-to-day activities and understanding of when and where interventions can be tailored for disrupting routines and modifying behavior change. This research aims to serve as a critical intermediary step in advancing the development of more effective interventions by exploring how context-specific strategies can be tailored to improve decision-making processes, ultimately making the adaptation of day-to-day routines easier and more automatic.

### Limitations of behavioral interventions

Behavioral interventions are often designed with a theoretical framework that emphasizes individual motivation, capability, and cognitive restraint for behavior change (Jacob et al., 2018; Liu et al., 2021; Room et al., 2017; Winter et al., 2016). For example, interventions using cognitive-behavioral strategies for weight management often target underlying motivation, beliefs, and emotions through cognitive restraint training that encourages slow and deliberate decision-making processes toward modifying eating behaviors (Amiri et al., 2020; Jacob et al., 2018). The Capability, Opportunity, and Motivation - Behaviour (COM-B) model is one approach used to inform the design of interventions by explaining how decisions about behaviors are driven by individual capability, opportunity, and motivation (Michie et al., 2011). Yet routine health behaviors are influenced by seemingly automatic decisions about day-to-day activities under constraints of limited time and resources (Betsch et al., 1998; Marteau et al., 2012). These automatic decisions are known as fast thinking and result in health behavior choices that are made unconsciously (Kahneman and Tversky, 1984). Health behaviors are not solely driven by intrinsic, reflective thought processes, and when executive functions are compromised from factors like fatigue, individuals commonly default to automatic decision-making processes that are influenced by environmental contexts and unconscious habits (Kahneman, 2003; Marteau et al., 2012).

Understanding the contexts that influence automatic decision-making processes is critical for tailoring intervention strategies so that behavior change techniques are individualized to the contexts in which patients make choices about their health activities. Strategies from other disciplines like behavioral economics provide insight into cognitive biases that influence automatic decision-making processes and strategies to improve decisions about engaging in health promoting activities.

### Contexts of decision-making processes

Behavioral economics is an evolving field, rooted in psychology and economics, that offers insights about cognitive biases that influence decision-making processes (Möllenkamp et al., 2019; Tversky and Kahneman, 1974; Vlaev et al., 2016). Behavioral economic principles have become widely applied within interventions and are distinct from behavioral science techniques (Gilovich et al., 2002; Kahneman and Tversky, 1984). Behavioral economic principles identify that individuals commonly default to heuristics, known as fast-thinking processes, that are automatic and emotionally charged and less commonly use slow, deliberate, and analytic thinking processes to make decisions day-to-day (Kahneman, 2003; Korteling et al., 2018). Nine of the most common cognitive principles that have been used to improve the design of behavioral interventions are outlined by the MINDSPACE framework including messenger, incentives, norms, defaults, salience, priming, affect, commitments, and ego (Dolan et al., 2015; Vlaev and Makki, 2018). A more detailed description of each principle within the MINDSPACE framework is outlined within Table 1.

**Table 1.** Description of MINDSPACE principles (Dolan et al., 2015; Vlaev and Makki, 2018).

Principle	Description
Messenger	Our perception of information is strongly shaped by the person delivering it. The influence of the messenger’s perceived authority, whether official or informal, significantly impacts us. When the expert shares demographic or behavioral similarities with the recipient, the effectiveness of the message is often enhanced. Additionally, our feelings toward the messenger play a crucial role. Beyond this, we also rely on more rational and cognitive processes to evaluate how persuasive the messenger is.
Incentives	Our reactions to incentives are influenced by predictable mental shortcuts. For instance, we tend to avoid losses more strongly than we pursue gains, a tendency known as loss aversion. The value we place on something often depends on our reference point. We also tend to overestimate small probabilities, which can make lotteries particularly motivating. Additionally, we mentally allocate money into specific categories, known as mental budgeting. Lastly, we exhibit a present bias, where we prefer immediate rewards over future benefits.
Norms	Our behavior is greatly influenced by the actions of those around us. Social and cultural norms are the expectations or rules that guide behavior within a society or group. These norms can be either explicitly stated or understood through observing others. People often form their understanding of social norms by watching how others behave. To effectively communicate norms, it’s important to connect them to your target audience and consider the impact of social networks.
Defaults	We tend to “go with the flow” when it comes to pre-set options. Many of our daily decisions involve a default choice, whether we realize it or not. Defaults are the options that are automatically selected if we don’t actively choose something else. These defaults have a strong influence, as people often stick with the default option, even when it carries significant consequences.
Salience	We are naturally attracted to what is new and seems relevant to us, and this focus greatly influences our behavior. People are more likely to notice stimuli that are novel (like messages in flashing lights), easily accessible (such as items placed near checkouts), simple (a catchy slogan), and personally relevant (especially during significant life changes like moving, starting university, or pregnancy). Additionally, we often rely on prominent anchors, such as unusual experiences, prices, or advice, to guide our decisions.
Priming	Our actions are frequently influenced by environmental cues without us even realizing it. When people are exposed to specific sights, words, or sensations, these cues can trigger related concepts in their memory, subtly altering their subsequent behavior. In other words, people may act differently if they’ve been “primed” by certain stimuli beforehand.
Affect	Our emotions strongly influence our actions. Emotional reactions to words, images, and events can be swift and automatic, often shaping our judgments more than conscious decisions do. People in a good mood tend to make overly optimistic judgments, while those in a bad mood are prone to making overly pessimistic ones.
Commitments	We strive to stay consistent with our public promises and to reciprocate the actions of others. Commitment devices help us achieve long-term goals, and research shows that commitments are more effective when the consequences of failure are higher. Making commitments public is a common way to raise these stakes, as breaking a public promise can harm one’s reputation. Additionally, the simple act of writing down a commitment taps into our strong instinct for reciprocity, which is closely tied to our sense of fairness.
Ego	We often act in ways that boost our self-esteem. We strive to maintain a positive and consistent self-image, and this extends to the groups we identify with. We also value being seen as consistent in our actions. However, when our behavior clashes with our self-beliefs, it’s usually our beliefs that shift to resolve the conflict, rather than our actions.

Changing default settings, peer comparison, and introducing incentives are the most widely applied behavioral economic principles that have shown to improve the effectiveness of behavioral interventions targeting healthcare professional and patient behaviors. Incentives are the most widely adopted behavioral economic principle used to target patient PA behaviors (Hare et al., 2021; Waddell et al., 2020). For example, patients who received a FitBit watch for monitoring PA and cash-based financial incentives engaged in significantly more minutes (29 minutes per week) of moderate to vigorous PA compared to patients that received a FitBit watch and charity-based financial incentive, or a FitBit watch alone (Finkelstein et al., 2016).

Interventions tailored to individual needs of patients (Santo and Redfern, 2020) and underlying motivation and cognitive restraint for behavior change (Jacob et al., 2018) do not adequately account for fast thinking and automatic habits that influence decisions about day-to-day health behaviors. Behavioral economic principles provide insight of contexts influencing automatic and fast thinking decisions however, the timing and location of patients’ day-to-day routines are critical for tailoring interventions by identifying opportunities to disrupt routines based on knowledge of when and where health activities occur. It is not evident how behavioral economic principles can be applied to tailor intervention strategies based on contexts of patients’ day-to-day routines and automatic decision-making processes for modifying behaviors.

### Aim and objectives

The aim of this study was to explore opportunities for incorporating context specific behavioral economic principles into patients’ PA routines. To achieve this aim, this study answers two more specific research objectives: (1) to identify contexts of facilitators and barriers influencing patients’ capability, opportunity and motivation to increasing PA; (2) to identify potential context specific behavioral economic principles that could be used to tailor secondary interventions for CVD.

## Methods

### Research setting

This study took place in the context of a voluntary cardiac prevention and rehabilitation intervention program for patients presenting risk factors for CVD, or patients that have recently experienced a cardiac event. A more detailed description of the research setting can be found in a previous publication (Barber et al., 2024b).

### Study design

This study used a qualitative descriptive approach to gather detailed information about patients’ day-to-day PA routines, including perceptions about factors that influence decisions about activities. Qualitative description is a particularly useful design to gather patients’ experiences and allows first person accounts to be described directly from those experiencing the phenomenon (Bradshaw et al., 2017; Kim et al., 2017; Neergaard et al., 2009).

### Patient recruitment and data collection

Purposeful recruitment of potential patients was conducted with patient cohorts enrolled in the cardiac program. Cohorts of 8–10 patients were purposively selected for recruitment if they were in week 2 or 3 of the 6-week program. Recruitment was carried out by a healthcare professional at the cardiac program by first approaching patients with information about the purpose of the study and what would be required to participate. The recruitment process was repeated with 5 different patient cohorts, with 6 patients agreeing to participate from each cohort, to generate a sample size sufficient for qualitative saturation (Hennink and Kaiser, 2022). To acknowledge the time commitment required for patients to be interviewed, every patient was offered an honorarium of a 25-dollar (CAD) gift certificate to a grocery store.

Interviews were conducted in-person by the first author at the cardiac program between June to September 2021. Interviews were structured to gather contextual information about patients’ activities, including what, where, and when routine activities occur, why they occur, relationships that influence activities, and perceptions about adapting routine activities. Patients were also asked to discuss their health goals and factors that support or prevent them from reaching health goals. Interview questions included, “How would you describe your typical activities on a given day?” “What sorts of activities do you do in your neighborhood?” “What are some of your health goals you are working toward?” “Are there times during the day you find it difficult to stay on track with your goals?” “How might your activities be influenced by who you are with?”

### Ethics approval

This study obtained ethics approval from Nova Scotia Health Research Ethics Board (REB#1026722). All patients provided informed consent for their de-identified and anonymous personal health information and direct quotes to be included in research results.

### Analysis

Interviews were audio-recorded, transcribed verbatim, and then coded by the first author with the aid of QSR NVivo12.6.1 qualitative data analysis software (QSR International, 2020). Deductive thematic analysis was employed (Fereday and Muir-Cochrane, 2006), guided by the COM-B model (Michie et al., 2011) and the MINDSPACE framework (Dolan et al., 2015; Vlaev and Makki, 2018) as coding frameworks to address the study’s research objectives. For the first objective, the COM-B model was applied to identify facilitators and barriers to increasing PA (Michie et al., 2011). A more detailed definition of each principle within the COM-B framework is provided in Table 2.

**Table 2.** Definition of COM-B constructs (Michie et al., 2011).

Construct	Definition
Physical capability	Skills, abilities, or proficiencies acquired through practice
Psychological capability	Knowledge, memory, attention, decision processes, behavioral regulation
Reflective motivation	Beliefs about capabilities and consequences, roles, identity, intentions, goals, optimism
Automatic motivation	Emotions, reinforcement such as rewards, incentives, punishment
Social opportunity	Social influences such as social pressure, norms, conformity, social comparisons
Physical opportunity	Environmental context and resources

For the second research objective, the MINDSPACE framework (Dolan et al., 2015; Vlaev and Makki, 2018) was used to explore patients’ cognitive biases and the potential application of context-specific behavioral economic techniques to improve decision-making processes toward increasing PA. Definitions of MINDSPACE principles are outlined in Table 1.

Data analysis followed a rigorous process of generating initial codes, searching for themes, reviewing themes, and defining and naming themes that represented patient responses (Braun and Clarke, 2006; Fereday and Muir-Cochrane, 2006). The COM-B model served as the coding structure for categorizing emergent themes as facilitators or barriers under each construct of capability, opportunity, and motivation. Similarly, the MINDSPACE framework guided the coding and thematic analysis of patient responses, linking emergent themes to specific behavioral economic principles. The first author (BVB) conducted the initial coding, and themes were collaboratively reviewed and refined with another research team member (MV). This iterative process ensured consistency and credibility in aligning patient responses with the theoretical frameworks, enabling a nuanced understanding of the factors influencing PA behaviors and opportunities for targeted intervention design.

## Results

A total of 58 interviews were conducted with 29 patients (19 men and 10 women). Two interviews were conducted with each patient, lasting on average 71 minutes (range 37–110 minutes). A total of 31 individuals were recruited to participate in this study, however, 2 dropped out of the study before the first interview due to lack of time to participate. A detailed description of patient characteristics has been previously published (Barber et al., 2024b) and a summary provided within Table 3.

**Table 3.** Description of patient characteristics.

Patient characteristics n %		
Age		
45–54	7	24%
55–64	7	24%
65–74	9	31%
75–84	6	21%
Sex		
Male	19	66%
Female	10	34%
Education level		
High school	5	18%
College/Trades	12	41%
University/Graduate degree	12	41%
Marital status		
Married/Common-law	22	76%
Single/Widow	7	24%
Household composition		
Alone	7	24%
With partner	15	52%
Partner and children	7	24%
Employment status		
Part-time/Retired working part-time	3	10%
Full-time	11	39%
Retired	12	41%
Sick leave – previous full-time	3	10%
Smoking status		
No	14	48.5%
Current smoker	1	3%
Previous smoker	14	48.5%
Reason for referral		
Prevention – no comorbidity	2	7%
Heart attack/Stroke – no comorbidity	14	48.5%
Heart attack – comorbidities	13	44.5%

**Figure 1.**
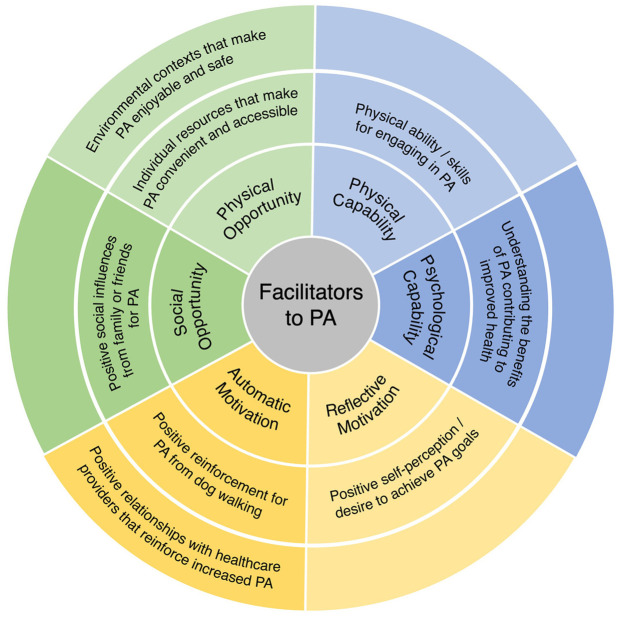
Facilitators to physical activity.

**Figure 2.**
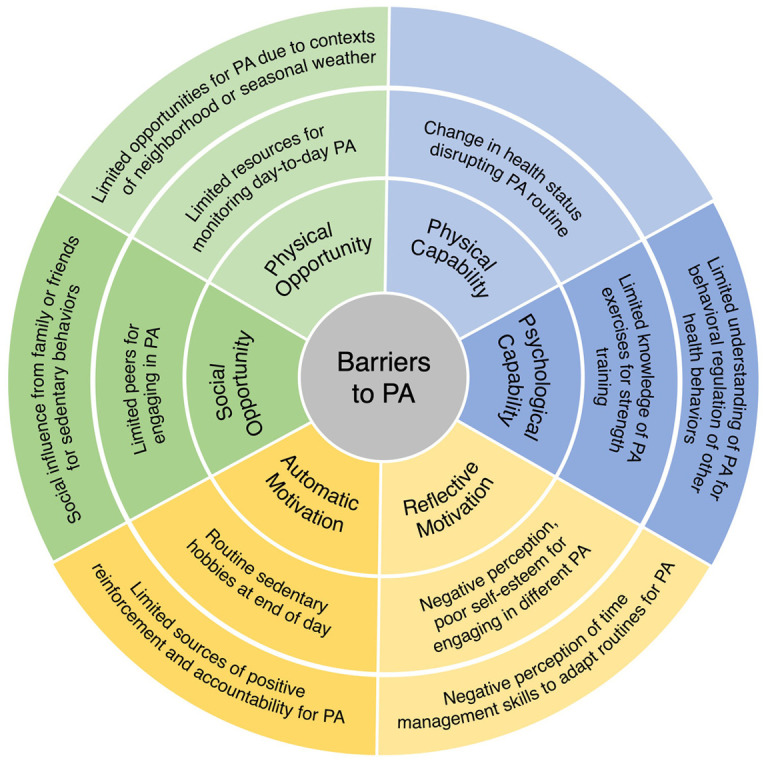
Barriers to physical activity.

**Figure 3.**
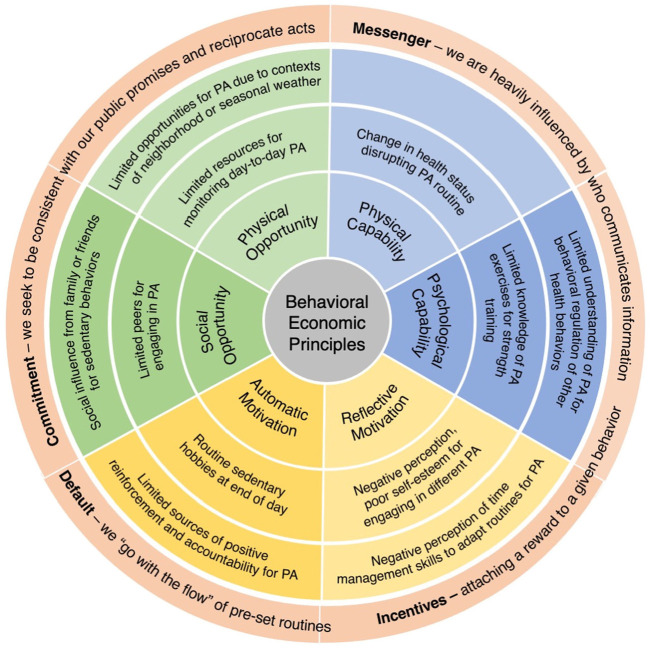
Behavioral economic principles.

 The findings are presented by drawing from patient accounts to describe: (1) facilitators and barriers to increasing PA, and (2) context specific behavioral economic principles for tailoring secondary interventions. Using deductive content analysis, patient-reported barriers and facilitators were categorized according to the constructs of capability, opportunity, and motivation within the COM-B model. A summary of these findings is presented in Figures 1 and 2. Themes aligned with behavioral economic principles from the MINDSPACE framework emerged during analysis, including disrupting default routines, increasing commitments, changing the messenger, and introducing incentives. The interconnections between COM-B-identified barriers to PA and the corresponding behavioral economic principles are visually represented in Figure 3.

### Facilitators to increasing physical activity

The following facilitators to PA are presented as health promoting abilities and knowledge, supportive social and environmental influences, and positive beliefs about capabilities and reinforcement (Table 4).

**Table 4.** Facilitators to physical activity.

COM-B construct description	Patient quote
Capability – physical	
* “At least three and a half hours, every day”* (P05) All patients discussed their physical ability and skills for engaging in PA every day.	As described by P11, “I run again. I do that every other day. Walking would be on the days I don’t run… Obviously we take kayaks out on the lake.”
Capability – psychological	
“Being more physically fit has no downsides.” (P02) Most patients (*n* = 22) described understanding the benefits of different PA contributing to improved health.	As P15 described, “I’m supposed to get my heart rate up for thirty minutes, five times a week. Just doing housework or gardening isn’t going to help me target specific areas, so I guess specific exercises with particular muscle groups.”
Opportunity – social	
* “I just wanted to see what yoga was all about, cause a lot of my friends are doing it”* (P27) Many patients (*n* = 21) discussed positive perceptions toward engaging in PA in connection to social influences from family or friends. Relationships with others often provided opportunities for patients to engage in PA, for instance spending time with friends or family who walk or play sports. Some patients described how observing others engage in PA positively influenced whether they would participate in the same activity.	Discussing the influence of their spouse, P17 described, “she’s better at exercising than I am… I’m thinking wow, can I do that? I’m trying to do it every day, even for ten minutes.”
Opportunity – physical	
* “We don’t want to walk out in the snow or ice anymore, so we bought a treadmill and both of us use it every day”* (P01) When discussing opportunities for PA, patients (*n* = 23) described the benefits of having resources that made opportunities for PA convenient and accessible on a regular basis. Individual resources included having equipment available at home or living within a building that provided resources for PA. Opportunities for PA were also discussed as resources within neighborhood environments that made it enjoyable and safe to engage in PA. Most patients (*n* = 26) described benefits of living close to parks or engaging in PA within their neighborhood.	Access to PA equipment was described by P20, “we’ve got three treadmills in our little gym, so I’ve learned how to use that…so if it’s going to rain … I’ll put my name on the sign-up sheet for the gym.” Having sidewalks around the neighborhood was described by P09, “to get out for my walk I really appreciate having sidewalks because otherwise it could be a dangerous road. It’s quite busy.”
Motivation – reflective	
* “I’m motivated to do more every week… I try to push myself… to be motivated you gotta want something”* (P19) All patients described positive self-perceptions, beliefs about capabilities, and desire to achieve PA goals. Patients described feeling capable of increasing PA within their routine and used strategies to stay motivated for reaching different PA goals.	Strategies for increasing PA were described by P13, “what I tried to do is use the time when I left… First day I tried it, I go around in half an hour. The next time I go down to twenty-five minutes. I did do it… Same distance, so I keep going a little faster every day.”
Motivation – automatic	
* “It has more weight coming from your doctor, I have a huge amount of trust”* (P06) Relationships with healthcare providers was perceived by most patients (*n* = 21) as a positive source of reinforcement for changing behaviors. When patients sought advice or were provided a recommendation, they described feeling an automatic incentive to make a change. Positive reinforcement for PA was also discussed when patients (*n* = 12) walked their dog. Patients described feeling motivated to get out for daily walks to take care of their dog and discussed the unlikelihood of walking as much if they did not have a dog.	When discussing why they walk more, P05 stated, “yes, my doctor asked me. I was doing two hours and she said, can you give me three hours? I’ve stuck to it… They have more invested in me than anybody else.” As illustrated by P07, “that’s really my exercise is walking the dog… But you don’t really have a choice with the dog. It’s only fair to her, she has to have a walk. The motivation is different because of her.”

### Barriers to increasing physical activity

The following barriers to PA are presented as limiting abilities and knowledge, restricting social and environmental influences, and negative perceptions and accountability (Table 5).

**Table 5.** Barriers to physical activity.

COM-B construct description	Patient quote
Capability – physical	
* “It’s my physical condition that prevents me from doing as much as I would like” (P24)* All patients described a change in health status as a barrier disrupting PA routines. Reduced physical capability for PA resulted from new or worsening health conditions, an acute injury, or physical limitations from reduced mobility.	Adjusting to an acute injury was discussed from P01, “so I used to walk six kilometers, and then it was knocked down to more like two. I had a bad injury for eight months… that really cut into my physical activity.”
Capability – psychological	
* “I was nervous to do anything strenuous, in terms of any weights”* (P15) Many patients (*n* = 22) described limited knowledge of strength training exercises, including what types of exercises were considered strength training and how strength training exercises could be integrated into their routine. Another barrier to increasing PA included limited understanding of PA for behavioral regulation of other health behaviors. Many patients (*n* = 27) described having poor diets while trying to increase PA.	When asked whether strength training was part of their routine P25 stated, “I don’t know I haven’t really thought… Arm, you know with the dumbbells, I don’t know, biceps and that sort of thing. Maybe sit ups. I’ve got to write it down what I plan to do.” As illustrated by P14, “I think I need to prioritize physical exercise… I’m plateaued… if I eat less, I’m gonna be starving to death.”
Opportunity – social	
* “They don’t really like to do exercises”* (P10) Social influences from family or friends to engage in sedentary behaviors was a common barrier discussed (*n* = 24). Opportunities for PA were limited from time spent during social activities with friends and family that were not interested in engaging in PA. Patients (*n* = 26) also described having limited peers to engage in PA as a barrier to increasing PA. Patients discussed most PA occurred alone and having peers would increase social opportunities for PA such as going for walks or trying new activities.	Spending time with a spouse that prefers to watch television was a barrier for P11, “she likes to sit down and watch TV…I’m probably more active when I’m on my own… if she’s gonna sit down, watch TV, so that we can spend some time together I’ll go and sit down with her.” As illustrated by P26, “I wish I had a couple of friends that were going to do the same thing or we could join to be sociable… I don’t know anybody; it means I have to go by myself.”
Opportunity – physical	
* “Well in winter I don’t walk as much, mainly because of the ice”* (P28) Limited resources for monitoring day-to-day PA were described as a barrier for increasing PA, such as monitoring intensity of activity. Patients (*n* = 15) discussed limited resources to monitor PA intensity and were unaware if they were staying within recommended heart rate targets. Limited opportunities for PA due to contexts of neighborhood or seasonal weather was also discussed by most patients (*n* = 24). Limiting opportunities for PA within neighborhoods included unsafe roads and parks for walking and hazards from ice and snow during winter months.	For P23, a barrier to increasing PA intensity at home was described as, “They would come over and do your heart rate and if I was pushing it too much they were watching. But doing that on my own, I don’t trust myself, I would just go too hard… It would be amazing if there was a button and I look at it and go okay, so this is what I’m at, because that’s too high. So, take it back.” As discussed by P17, “I don’t walk much… It’s not a good road to walk on, it’s very narrow shoulders. It’s actually dangerous.”
Motivation – reflective	
* “I’m a skinny white guy, you sort of get that bias or thought in your head”* (P02) Negative perceptions and poor self-esteem for engaging in different PA was discussed as a barrier for increasing PA (*n* = 23). Patients described limiting beliefs about capability to engage in different PA based on negative self-perceptions of age and appearance. Patients also described negative perceptions of their capability for managing time to adapt routines for increased PA. Patients discussed scheduling PA around their existing routines and were challenged with identifying opportunities to adapt routines for PA.	Attending an exercise class geared for seniors was important for P09, “the main thing that I would think about whether or not I could keep up with that class, because if you don’t all have the same issues then they’re waiting, and I would annoy them.” As illustrated by P03, “unless it’s integrated into other things that my family is doing… I get to be motivated to fit it in and when I was younger, it was nothing to do something at nine o’clock at night… well, it’s not that simple anymore.”
Motivation – automatic	
*“Watch TV, I don’t have much more to do”* (P13) Sedentary hobbies at the end of the day were described by all patients (*n* = 29) as a barrier for increasing PA. Watching television, playing cards, or reading were described as examples of sedentary activities part of patients’ routine in the evening. Limited sources of positive reinforcement and accountability for PA was another barrier that made it challenging to maintain motivation for increased PA. Patients (*n* = 19) described limited sources of external motivation, such as positive reinforcement from healthcare professionals or accountability from family and friends.	As described by P12, “if I can read, with nothing else to do, I can spend all afternoon reading. In the evening if when I watch TV, any time after seven p.m.” Limited sources of positive reinforcement were described by P29 as, “I’ll go do it at a gym with someone over me or just nodding… My wife or someone checking in at the gym, I think positive reinforcement goes a long way for me.”

### Behavioral economic principles

The following behavioral economic principles are presented as strategies to support automatic decisions toward increasing PA like disrupting default routines, increasing commitments, changing the messenger, and introducing incentives (Table 6).

**Table 6.** Behavioral economic principles.

Principle description	Patient quote
Changing the default	
* “It’s really easy to just do the routine”* (P15) Disrupting default habits were discussed as a potential strategy for increasing PA. Adapting default routines and changing perceptions that PA required a pre-set amount of time were described as ways patients could break free from default routines and identify opportunities for PA.	Breaking free from default routines was described by P15, “The problem is that if you’re not thinking about it all the time, you’re really intentional, it’s really easy to just do the routine of, you know, go in here and do whatever and then shoot I didn’t get my walk in today… Like if I’m thinking about my day and when I do, I find that works. Like when am I going to work in some exercise, will that be at the gym next door? Will it be walking at lunchtime? … I’ll have to be more intentional.” The perception that PA requires a pre-set amount of time was described by P11, “when you think of it as a time thing, and ten minutes, obviously that makes it sound easier. So, already I’m thinking like it’s easy, I could fit ten minutes into my schedule as it is, by just being more efficient in the mornings.”
Increasing commitments	
*“If you signed up to do something, there’s more of a commitment”* (P02) Sources of commitment were described as potential strategies for increasing PA. Signing up for an exercise class, scheduling PA time, and accountability of public promises for PA were described as intrinsic and extrinsic commitments that could influence opportunities for increased PA.	Committing to an exercise class was described by P02, “It does matter. I think there is. If you signed up to do something, there’s more of a commitment, saying okay, I’ve committed to this. It’s at eight o’clock. I got to be there, versus saying yeah you know tonight I’ll go walking. But then you know something comes up it’s easier to slow down. I think anything that you sort of committed to that is outside, it’s definitely more important to do that.” Commitments were also described as being accountable to public promises, as P06 stated, “I think it definitely helps because it’s like they say when you have a goal, you should tell someone. It makes you sort of accountable to them, even though you may not be, just because you share that goal publicly, you kind of go. Okay I said I was gonna walk 10000 steps a day, so I better get up and do it. I think there’s a lot of power in that.”
Changing the messenger	
*“Having a healthcare provider check in on my results…that would be enough for me”* (P20) Individualized messages from healthcare professionals were described as potential strategies for increasing PA. Receiving information about adapting health activities and having follow-up messages to monitor results of health tests were described as opportunities that would influence increased PA.	Regular communication with a healthcare professional was described by P20, “having to give her my results and maybe having a check-in done… I think for me the benefit is, and I don’t know whether it’s because I’m single. I live alone and don’t have somebody in my sphere… I think to me that would be more than enough to motivate me to do things.” The influence of a healthcare provider following-up on test results and monitoring changes in behavior was described by P05, “I’m looking to see the blood stuff, your records. And how each thing, you know, your lipids are at this level… It’s the little pieces of the puzzle trying to get people to change their whole lifestyle. That’s not gonna happen. Taking little pieces of your lifestyle, and I think by doing that individually. You’re better when someone calls you, that’s my feeling.”
Introducing incentives	
*“Everybody likes rewards, don’t matter whether you’re a kid or not, to be told you’re doing a good job”* (P04) Incentives from achieving individualized PA goals was described as a potential strategy that would influence increased PA. Thirteen patients that used a digital health watch to monitor PA described incentives as a potentially useful strategy to individualize a PA goal. Sixteen patients that did not use technology or a digital watch to monitor PA described incentives as a potentially useful strategy, but discussed the importance of intrinsic motivation to drive increased PA.	The influence of incentives was described by P21 as, “I would have no problem with doing that, I wouldn’t expect a reward at the end, my reward would be myself. I met the goal or close to the goal.” Using incentives as a gamification strategy was described by P02 as, “you know the whole gamer theory. I find that even sort of incentivizes me… I think it would have value in and of itself would be the reason I would do it.”

## Discussion

Contexts that influence patients day-to-day decision-making processes provide important insight for identifying potential behavioral economic principles that could be used to influence seemingly automatic decisions about increasing PA. Results from this study highlight patients had capability and were motivated for increasing PA, but were challenged with identifying opportunities, such as when, where, and how they could adapt day-to-day routines to increase PA. Facilitators to PA are important for understanding patient capability for PA, such as where patients are at along different stages of readiness to change (Mauriello et al., 2017). Barriers to PA provide insight of contextual factors influencing decisions about PA, such as patients’ perceptions of intrinsic and extrinsic constraints influencing opportunities for increasing PA. Although the COM-B model is useful for understanding individual drivers of behavior, the COM-B model is not useful for identifying factors that influence common automatic decisions about day-to-day activities. The integration of behavioral economic principles is critical for understanding patients’ cognitive biases and identifying potential strategies that can support automatic decisions toward increasing PA like disrupting default routines of watching television, increasing commitments by signing up for exercise classes, changing the messenger with texts from healthcare professionals, and introducing incentives to reach daily step count goals. The use of behavioral economic principles as a coding framework provides useful information about tailoring strategies to the spatiotemporal contexts of patients’ automatic decision-making processes. For example, timing and location sequences of patient routine activities provide import information of when and where patients are challenged with task-switching, such as disrupting automatic sedentary routines of watching television after dinner. Future research should continue to explore suitability and efficacy of behavioral economic principles before they are integrated into an intervention to ensure strategies are tailored to the contexts of barriers influencing patients’ cognitive biases and resources and opportunities of different patient populations for reaching behavioral goals.

This study represents a crucial first step in enhancing behavioral interventions by identifying context specific opportunities that make decisions for adapting routines easier and more automatic. Behavioral economic principles are one example of intervention strategies that target fast-paced decision-making processes, such as adding environmental cues to support development of routines that are habitual and automatic (Marteau et al., 2012). Framing, incentives, and social norms are some of the most applied behavioral economic principles within interventions for PA (Blaga et al., 2018; Hare et al., 2021; Waddell et al., 2020). For example, a Nintendo Wii video game using threat-framed messages has shown to improve positive PA attitudes, self-efficacy, and perceived behavioral control among adolescents (Lwin and Malik, 2014). Furthermore, the use of incentives has shown to increase population-wide PA for users of the Carrot Rewards mobile app in British Columbia and Newfoundland and Labrador, Canada (Mitchell et al., 2018, 2020). Our study strengthens the evidence base supporting incentives as an appealing intervention strategy for achieving daily PA goals. However, more than half of patients (*n* = 16) described their intrinsic motivation for PA and did not perceive that an incentive was necessary to initiate a change in behavior. Although patients had positive perceptions of their motivation and capability for PA, our study patients did not reflect on how their motivation and capability for PA fluctuated throughout the day, such as when they were more likely to default to sedentary activities. When executive functions are fatigued, and patients have diminished self-regulating control for making goal-directed decisions about their behaviors; incentives may be critical for encouraging automatic decisions about adapting routines to reach an individualized goal for PA before the end of the day. Incentives have shown to positively influence PA (Barte and Wendel-Vos, 2017); however, it is less evident whether incentives have been tailored using information about the timing and location of behaviors, such as incentivizing increased PA during times when patients are prone to defaulting to sedentary activities.

### Tailoring individualized interventions

The significance of applying behavioral economic principles within this study was to understand the contexts influencing decision-making processes and identify what potential strategies could be applied to tailor interventions to the individual needs and health goals of patients. Results from this study provide insight of the importance of tailoring digital tools to the common decisions patients make as part of their daily routines. For example, information that patients are challenged by limited knowledge of PA exercises for strength training provides an opportunity for healthcare professionals to work with patients to develop their own intervention strategies like scheduling calendar notifications with instructions of different strength training exercises. Calendar notifications could be tailored and delivered at the most opportune time and place based on patients’ existing day-to-day routines, access to equipment, and capability for engaging in different strength training exercises.

Although digital health tools provide a means of delivering behavior change techniques, it is unclear whether technology-based strategies would have the same effectiveness for all patients. For example, 13 patients within this study were using digital tools, including watches or mobile applications for self-monitoring PA, and 16 patients were not using any technology for self-monitoring PA. It is unlikely digital tools used to deliver behavioral economic intervention strategies would have the same impact on patients that regularly interact with technology-based stimuli versus patients that do not use technology. For example, a meta-analysis of interventions using prompts to increase patient engagement with technology-based interventions found small-to-moderate positive effects that prompting reminders increased patient engagement in technology-based interventions in comparison to no prompting strategy (Alkhaldi et al., 2016). To increase effectiveness of interventions using digital tools it is critical interventions are flexible enough to adapt behavior change techniques, so they are specific to individual needs and preferences. For example, interventions could be tailored by delivering individualized counseling via mobile text messages with patients that have high engagement with using technology tools versus offering counseling via phone calls with patients that have low engagement with using technology tools. CVD interventions using mobile text messages to send motivational and educational information that encourage specific PA goals have shown to significantly increase peak aerobic capacity from baseline after 24 weeks post-intervention (Frederix et al., 2015) and increase moderate to vigorous PA minutes (>105 minutes/week) from baseline after 2 weeks post intervention (Legler et al., 2020). Additional research is required to assess long-term effectiveness of digital health tools such as identifying whether digital health tools lose novelty when patients are exposed to the same stimuli over time (Mobekk et al., 2020).

Individualizing digital health tools is also critical for tailoring interventions across diverse patient populations that experience unique facilitators and barriers to behavior change, including chronic time deficits and limited knowledge of self-management strategies (McQuoid et al., 2017; Stuart-Shor et al., 2012). CVD disproportionately affects marginalized populations (Mensah, 2018), including African Nova Scotian populations within communities where this study took place (Kisely et al., 2008). It is not evident how behavioral interventions are adapted to target recruitment of diverse populations and what contextually relevant strategies are used to meet the needs of diverse and marginalized populations (Stuart-Shor et al., 2012). Research methods used in this study could be applied to advance equity, diversity, and inclusion goals by adapting the design of interventions specific to the contexts of diverse populations and community settings. For example, patient populations that experience chronic time deficits are unlikely to participate in a clinic-based intervention (Stuart-Shor et al., 2012), however, digital technologies provide a means of delivering interventions remotely using individualized health education and counseling strategies. By integrating geo-ethnographic techniques (Barber et al., 2024a, 2024b), interventions could gather information of patients’ time-use patterns and tailor strategies to the unique social and environmental contexts influencing opportunities for adapting health behaviors. For example, patients challenged by lack of transportation may be encouraged to participate in an intervention using incentives for daily PA goals, such as encouraging patients to walk to a public transportation stop further away from home. Incentive strategies could also be tailored to encourage patients to watch educational health videos during opportune times when commuting on public transportation.

### Integrating strategies from other disciplines

Improving the effectiveness of behavioral interventions to prevent chronic disease and associated risk factors will require evidence from other disciplines and methodologies (Bacon et al., 2020; Nieuwlaat et al., 2013). This study explored why behavioral economic principles are important for understanding contexts influencing decision-making processes and what strategies are potentially useful for improving automatic decisions toward increasing PA. Behavioral economic principles have not yet, to our knowledge, been integrated with geo-spatial contexts to identify the most opportune time and place to deliver behavioral economic principles. When geo-ethnographic techniques and behavioral economic principles are combined, the effectiveness of behavioral interventions can be strengthened by identifying where, when, and how to disrupt routine activities with context specific strategies that improve automatic decisions to increase PA. The sustainability of intervention strategies will depend upon using information about the timing and place of existing routines to support decisions about adapting health activities that are easy and automatic.

### Limitations

A limitation of this study was gathering patient perceptions about their decision-making processes without testing whether the identified behavioral economic principles are effective for increasing PA. Future research should consider integrating the APEASE framework to assess implementation of behavioral economic principles and factors influencing uptake of intervention strategies within the contexts of clinic-based interventions (Michie et al., 2011). The APEASE criteria outline important context-based decisions that need to be considered when adapting interventions including: (1) affordability, (2) practicality, (3) effectiveness and cost-effectiveness, (4) acceptability, (5) side-effects/safety, and (6) equity (Michie et al., 2011). Many behavioral economic principles are cost-effective, however, without considering the contexts of existing health interventions, including human and financial resources available, the feasibility of implementing novel intervention strategies may be overlooked.

### Conclusion

Coupling behavioral economic principles with spatiotemporal contexts of patient day-to-day routines provides critical information of what behavioral economic principles can be tailored based on knowledge of when and where patients’ health activities occur. This study explores facilitators and barriers to increasing PA and how behavioral economic principles could potentially be used to tailor secondary interventions for CVD. Results indicate patients were capable and motivated for PA, but were challenged with identifying opportunities for adapting routines to increase PA. Disrupting default routines, increasing commitments, changing the messenger, and introducing incentives were identified as potential behavioral economic principles that could be applied to influence patient decision-making processes for increasing PA. Further research is required to implement and test the use of behavioral economic principles identified in this study to explore whether the identified methods are effective at improving the design of CVD interventions.
